# Sources of drug information and their influence on the prescribing behaviour of doctors in a teaching hospital in Ibadan, Nigeria

**DOI:** 10.4314/pamj.v9i1.71188

**Published:** 2011-06-03

**Authors:** Kazeem Adeola Oshikoya, Ibrahim Oreagba, Olayinka Adeyemi

**Affiliations:** 1 Pharmacology Department, Lagos State University College of Medicine, Ikeja, Lagos, Nigeria; 2 Pharmacology Department, College of Medicine, University of Lagos, Idiaraba, Lagos, Nigeria

**Keywords:** Drug, prescription, information source, promotion, pharmaceutical company, influence, doctors, Nigeria

## Abstract

**Background:**

Pharmaceutical drug promotion is a means of informing health professionals about new drugs. The approach is often times unethical and inappropriate and may promote irrational prescribing. Dearth of information on impact of pharmaceutical drug promotion on prescribing behaviour of doctors in developing African countries has necessitated this study. We therefore aimed to determine the sources of drug information for doctors working in a teaching hospital in Nigeria and to assess the self-reported impact of the sources on their prescribing behaviour.

**Methods:**

A total of 163 doctors working at the University College Hospital (UCH), Ibadan in Nigeria were evaluated with a questionnaire for their demographics and sources of drug information. For doctors who relied on drug promotion, they were asked to self-report and self-rate their opinion on extent of interactions with pharmaceutical companies as well as how such interactions had impacted on their prescribing behaviour. Apart from the demographics, each question was evaluated with a typical five-level Likert item. Data analyses were with simple descriptive statistics.

**Results:**

Of the 400 doctors working at UCH, only 40.8% participated in the study. Drug information was sourced from colleagues (161, 98.8%), reference books (158, 96.9%), pharmaceutical sales representatives-PSRs (152, 93.2%), promotion materials (151, 92.6%), scientific papers/journals/internet (149, 91.4%), and drug promotion forum/product launches (144, 88.3%). Each source was highly utilized but there was no wide variation in their pattern of use. According to the self-report of over a half of the respondents, PSRs was an accurate and reliable drug information resource; PSRs increased their awareness of the promoted drugs; and their prescribing behaviours were influenced by information from PSRs.

**Conclusion:**

Respondents tend to rely on a broad range of drug information resources which include potentially inappropriate resources such as PSRs. Since this study was based on self-report, the influence of drug information resources reported by the respondents on their prescribing behaviour may have been underestimated. Measures should be taken to minimize interactions between PSRs and the respondents.

## Background

Irrational prescription is a public health problem with the potential to harm both the individual and society. It is, possibly a contributory factor to the increasing pharmaceutical expenditure world-wide. Drug expenditure is a major concern for policy makers in Europe and has prompted them to suggest healthcare reforms [1].

Several factors have been identified to influence doctors’ prescribing decisions and practice [2–4]. Some factors such as the physicians’ age and gender, their training, the socio-economic characteristics of the practicing environment, and the healthcare demand are fixed and may not offer much opportunity for modification and improvements in prescribing behaviour [5, 6]. However, other factors including physicians’ level of education and experience, frequency of visits by pharmaceutical sales representatives (PSRs), number of patients examined per day, and various social factors are amenable to change and can be modified to improve physicians’ prescribing behaviour [7, 8].

Disease mongering is a potential means of creating an enormous market for drugs [9] but its influence on the prescribing behaviour of physicians has not been explored. Many normal life processes like birth, ageing, sexuality, unhappiness and death have been medicalised and are promoted as illnesses by the pharmaceutical industry [10]. Opinion leaders from the medical profession are used by the pharmaceutical industry to lure doctors to prescribe medicines for normal life processes when infact none is required [11].

Drugs play an important role in the treatment of ill patients. In Nigeria, drugs are prescribed to more than 60 percent of the patients that consult with doctors [12, 13]. PSRs are frequently the only source of information about medicines in developing countries where there may be as many as one representative for every five doctors [14].

The World Health Organization (WHO) defines pharmaceutical promotion as “all information and persuasive activities by manufacturers and distributors, the effect of which is to induce the prescription, supply, purchase and/ or use of medicinal drugs” [15]. The WHO and some NGOs are bothered about the unethical and inappropriate approach to the promotion of pharmaceutical products. At the 1997 roundtable on WHO's Ethical Criteria for Medicinal Drug Promotion there was firm agreement that inappropriate promotion of medicinal drugs remains a problem both in developing and developed countries [16]. Alongside the concern for unethical and inappropriate drug promotion, there is also increasing concern over irrational, inappropriate, or sometimes even harmful prescribing [17, 18].

Given the above problems and knowing that pharmaceutical companies play active roles in marketing their products, it is important to investigate how much influence these companies have on the prescribing behaviours of healthcare practitioners in developing African countries. Unfortunately, there is limited information on research in this area in Nigeria. This study is therefore aimed at determining the sources of drug information for doctors working in a teaching hospital in Nigeria and to assess the self-reported impact of the sources on their prescribing behaviour.

## Methods

### Study population

This was a prospective study involving doctors working at the University College Hospital (UCH), Ibadan, Oyo State in Nigeria. UCH is a premier teaching hospital in South-western Nigeria. Approximately four hundred doctors are in the employment of this hospital. They are distributed into thirteen departments namely: internal medicine, surgery, paediatrics, family medicine, obstetrics and gynaecology, ophthalmology, otorhinolaryngology, radiology, radiotherapy, dentistry, community health, accident and emergency, orthopedics and trauma. Due to the small number of doctors in some departments and for the convenience of sample analysis, internal medicine and accident and emergency departments were grouped together as general medicine. Similarly, surgery, ortorhinolaryngology, and orthopedics and trauma departments were grouped together as surgery specialties. Only seven of these departments (general medicine, surgery specialties, paediatrics, family medicine, obstetrics and gynaecology, ophthalmology, and radiotherapy) were selected for this study. Other departments were excluded either because drugs were not routinely prescribed or because only a few varieties of drugs were routinely prescribed. Thus, the PSRs are likely to interact less with doctors from the excluded departments.

### Data collection

A questionnaire was developed by the authors from previous studies that evaluated the influence of drug promotion on prescribing practices of doctors in developed [19, 20] and developing countries [21, 22], specifically for the purposes of this study.

The questionnaire was in three sections. The first section focused on the demographics of the doctors; the second section asked doctors to indicate their response to a series of statements about their sources of information for prescribing and extent of their interactions with PSRs, in each case a typical five-level Likert item was used. The third section focused on the perception of doctors on the reliability of PSRs and pharmaceutical materials as a source of prescribing information and how these factors would influence their prescribing behaviour. This last section also contains series of statements requiring responses using the five-level Likert item as it applied to the second section.

The questionnaire was piloted among 10 doctors at the Lagos University Teaching Hospital (LUTH), Lagos, Nigeria and, after minor modifications; it was administered to the study population at UCH. Only the doctors who were willing to voluntarily participate were recruited for the study. The questionnaire was allowed a maximum of one week with each participant so as to allow enough time for the filling. In order to increase the response rates, the participants who were unable to produce the previous copy of the questionnaire were given a new one to be filled-in and returned on the spot. All the questionnaires were completed anonymously. Confidentiality of the information tendered was assured to the participants.

The ethics committee of UCH Ibadan approved the study.

### Data analysis

The data obtained were analysed with SPSS version 15. Results were presented as median with inter-quartile range (IQR) for time related variables and as frequencies, percentages, and pictorial diagrams for responses to the questions in Likert-items.

## Results

**Figure 1:**
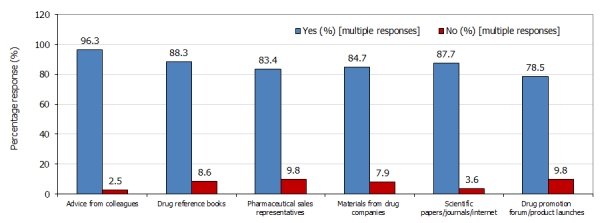
Pattern of use of drug information sources in a group of doctors in a teaching hospital in Ibadan, Nigeria

One hundred and sixty three (40.8%) of the 400 doctors working at the UCH Ibadan participated in the study. The characteristic features of the respondents are shown in Table 1. Responses to the second part of the questionnaire showed that all the doctors obtained drug information from more than one source which may explain the multiple responses to each of the questions. Of the 163 respondents, drug information was sourced from colleagues (161, 98.8%), drug reference books (158, 96.9%), PSRs (152, 93.2%), materials from drug companies (151, 92.6%), scientific papers/journals/internet (149, 91.4%), and drug promotion forum/product launches (144, 88.3%). The pattern of use of the drug information sources is presented in Figure 1. Although all the sources were highly utilized, there was no wide variation in their pattern of use. Seeking drug advice from colleagues was the most highly utilized source.

**Table 1:** The demographics and characteristic features of the respondents

Characteristics	Values
Median age (years)	36 (IQR 32–48)
Median year of practice in a teaching hospital (years)	6.9 (IQR 1.6- 16.5)
Male: female ratio	1.6: 1
	
**Cadre**	
House officers	24 (14.7%)
Medical officers	22 (13.5%)
Junior residents	45 (27.6%)
Senior residents	48 (29.4%)
Consultants	24 (14.7%)
	
**Area of specialization**	
General medicine	38 (23.4%)
Obstetrics and gynaecology	31 (19.0%)
Family medicine	29 (17.8%)
Surgery specialties	24 (18.4%)
Paediatrics	17 (10.4%)
Ophthalmology	11 (6.7%)
Radiotherapy	7 (4.3%)
IQR: inter Quartile range	

**Figure 2:**
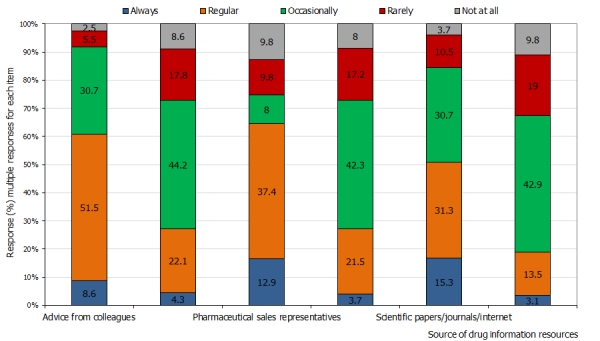
Frequency of use of drug information sources in a group of doctors in a teaching hospital in Ibadan, Nigeria

Amongst the respondents who utilized each of the drug information sources, their frequency of use is as indicated in Figure 2. A few respondents always rely on drug information from colleagues, materials from drug companies, or drug promotion/product launches. Over a half of the respondents regularly rely on their colleagues. Approximately 40% of the respondents occasionally rely on drug reference books, materials from dug companies, or drug promotion/product launches for prescribing information. Less than 10% of the respondents rarely consult their colleagues for prescribing information. There appears to be a wide variation in the frequency of use of the information sources.

Multiple responses to the statements in the third section of the questionnaire were also observed in this study. This section assessed the impact of drug promotion by PSRs on the prescribing behaviours of the respondents. Their responses are as indicated in Table 2. Over a half of the respondents agreed that PSRs are efficient sources of drug information; accurate and reliable drug information can be sourced from PSRs; drug promotion by PSRs, during their detailing, do increase respondents’ awareness of the promoted drugs; their prescribing behaviours were influenced by drug information obtained from PSRs, this information was considered very useful and were readily used when prescribing. This group of respondents also agreed to prescribing promoted drugs if convinced of the information provided by the PSRs on the drug benefits. Approximately one tenth of the respondents strongly agreed to most of the statements on impact of drug promotion on their prescribing behaviours.

**Table 2:** Impact of drug promotion on the prescribing behaviours of a group doctors in a teaching hospital in Ibadan, Nigeria

2	Responses (n=163)				
	
Statements	Strongly agree (%)	Agree (%)	Undecided (%)	Strongly disagree (%)	Disagree (%)
PSRs is an efficient source of drug information	17.2	52.8	18.4	9.8	1.8
Drug information from PSRs is accurate and reliable	12.3	53.4	23.3	9.2	1.8
Detailing of a PSR increases my awareness of promoted drug	16.6	65.6	12.9	3.7	1.2
Detailing of a PSR increases my preference for prescribing the promoted drug	13.5	46.0	29.4	9.2	1.8
Drug information from PSRs influenced my informed decision to prescribe	13.5	58.9	20.9	4.3	2.5
If convinced of a drug benefits by PSRs, I will prescribe to patients	22.1	60.7	9.2	6.1	1.8
Drug information from PSRs is often irrelevant	1.8	10.4	22.1	50.3	15.3
PSRs are good sources of drug information but rely less on them when prescribing	8.6	33.7	25.2	5.2	7.4
PSRs induce me to prescribe branded drugs even when generics are available	15.3	21.5	20.2	27.6	15.3
Drug information from other sources more important and reliable than from PSRs	11.7	33.1	22.7	27.0	5.5
Drug information from PSRs is usually useful and readily used when prescribing	16.6	52.8	21.5	6.7	2.5

PSR: pharmaceutical sales representatives

## Discussion

Less than half of the doctors working at UCH participated in the study. This was probably due to pressure of work on the doctors which did not allow them the time to attend to the questionnaire. However, the relatively small population size (163) of the doctors who participated in this study is similar to the population (137 and 185 doctors) surveyed in other studies from North central and South-eastern Nigeria, respectively [21, 23].

The doctors relied on multiple sources of drug information as indicated by their high responses to each of the questions [Figure 1]. Similar pattern of use of drug information resources has been reported among physicians in Greece and Cyprus [19], Australia [24], Ireland [25], Turkey [8], Nigeria [21], and Pakistan [26]. PSRs and promotional materials, as a source of drug information, may give new information about drugs; the benefits, efficacy and safety of such drugs may be exaggerated while their adverse effects downplayed [20, 27]. The information contained in promotional materials and from the PSRs may or may not be as accurate as it appears [28]. Reliance on pharmaceutical promotions may therefore promote improper, inappropriate and undesirable prescribing practices [29, 30].

Over half of the doctors would regularly seek drug information from their colleagues compared to less than a half that would occasionally consult reference books. Previous studies have shown that reliance on colleagues was the most highly ranked information source preference by rural and urban primary care doctors in the United States of America [31, 32] and the General Practitioners in Ireland [25].This practice was referred to by Williamson as a preference for human sources rather than paper sources [33].The costs associated with acquiring information from reference books, internet and journals are likely to be bore by individual doctors in resource poor countries like Nigeria and may be more of a burden to the doctors. This may explain why the respondents rely more on human drug information source which attracts no cost. It should be recognised that in a self-report study like this, the respondents are likely to rate their colleagues highly as a source of drug information but in practice, other information sources may be highly utilised. This is however a limitation of self-report studies [25].

Over 40% of the respondents occasionally rely on information from pharmaceutical companies or drug promotion forum/product launches. Reliance majorly on these sources for drug information is very rampant in many developing [21, 23, 26] and some developed countries [24, 34]. These materials can be highly informative as long as they are critically appraised [35]. Their use without appropriate appraisal may promote irrational prescribing. In Nigeria, doctors may find it difficult to discuss the validity of drug information in promotional materials because critical appraisal of such information is not taught in medical schools. Thus, integrating teaching of ethical issues surrounding drug promotion and critical appraisal of promotion materials into medical curriculum, and proper assessment of students; both theoretically and practically, may reduce the extent of doctors’ reliance on promotional materials and PSRs for accurate drug information.

Another limitation of a self-report study is underestimation of the effect of pharmaceutical promotion on the prescribing behaviour of doctors which are recognised limitations of the previous studies [8, 17]. In our study, over 50% of the respondents self-reported that PSRs was a good, accurate and reliable drug information source. This finding may however differ in practice. It may however explain why many of the respondents found information from PSRs very useful and ready to use when prescribing. This further emphasizes the need to teach critical appraisal of drug information from pharmaceutical industry as a potentially useful information resource. Educational intervention programmes on rational drug use may therefore be necessary to provide a framework for prescribers to objectively assess information provided by PSRs before deciding to prescribe their products. Teaching medical students how to appropriately interact with PSRs has been reported to impact positively on their prescribing attitudes [36].

Doctors may not be aware of how much they are exposed to drug promotion, thus a self-report study like this may not have appropriately assessed the impact of drug promotion on prescribing behaviour of the doctors. Further studies that would establish causal relationships between exposure to drug promotion and the prescribing habits of the doctors are therefore suggested. The small study population and focusing on only one institution in Nigeria may limit the generalizability of our results. It is to be hoped that a national survey that will involve doctors from many hospitals in Nigeria would address these problems in the future.

## Conclusion

Reliance on a wide range drug information resource is commonly practiced by the respondents. The use of potentially inappropriate information resources remains a major challenge facing the respondents and of concern for rational prescribing. Interventions in the form of continuous medical education may be required to improve their information-seeking behaviours. It is self-reported by the respondents that pharmaceutical companies influenced their prescribing behaviour. Measures may be necessary to minimize interactions between PSRs and the respondents.
